# Annie Laurie

**Published:** 1883-09

**Authors:** 


### ANNIE LAURIE.

THE song of "Annie Laurie" was written by William Douglass, of Fingland, in the stewartry of Kirkcudbright, hero of the song "Willie was a Wanton Wag." Annie Laurie was the eldest of the three daughters of Sir Robert Laurie, of Maxwelltown. Her birth is thus recorded by her father in the family register: "At the pleasure of the Almighty God, my daughter, Annie Laurie, was born on the 16th day of December, 1682 years, about 6 o'clock in the morning, and was baptized by Mr. Geo." (Hunter, of Glencairn). From the curious collections of manuscripts left by Mr. W. F. H. Arundel, Barjary Tower, Dumfrieshire. we learn that Annie was wooed by Douglass, and that she refused him. He then made a runaway match with a Miss Elizabeth Clark, of Glenborg, in Galloway. In 1709 Annie married James Fergusson, of Craigdarrook, and was the mother of Alexander Fergusson, the hero of Burns' song, "The Whistle." James Grant, in his novel, "Walter Fenton; or the Scottish Cavalier," gives a full account of the composition of "Annie Laurie." The early version of the song is as follows:

Maxwellton's banks are bonnie,
Where early fa's the dew;
Where me and Annie Laurie
Made up the promise true!
Made up the promise true,
And never forget will I,
And for bonnie Annie Laurie
I'll lay me doon and dee.

She's backit like the peacock,
She's breastit like the swan;
She's jimp about the middle,
Her waist ye weel micht span;
Her waist ye weel micht span,
And she has a rolling eye;
And for bonnie Annie Laurie
I'll lay me doon and dee.

The most touching story connected with the song is that told in Bayard Taylor's "Crimean Incidents":

They lay along the battery's side,
Below the smoking cannon.
Brave hearts from Severn and from Clyde,
And from the banks of Shannon.

And there, in sight of the Redan and the Malakoff, with to-morrow's battle coming some one called for a song, and—

They sang of love and not of fame.
Forgotten was Britain's glory;
Each heart recalled a different name,
But all sang "Annie Laurie."

Dear girl! her name he dared not speak,
But as the song grew louder
Something upon the soldier's cheek
Washed off the stains of powder.

